# Supplementary material for: Association between optical coherence tomography and functionally severe stenosis assessed by quantitative flow ratio in coronary intermediate lesions
Source: PLoS One. 2025 Jun 10;20(6):e0324872. doi: 10.1371/journal.pone.0324872 (PMC12151369; doi:10.1371/journal.pone.0324872)
Supplement: Attachment — Submitted filename: Review Comments.docx [file pone.0324872.s002.docx]

Review Comments

Title: Association between optical coherence tomography and functionally severe stenosis assessed by quantitative flow ratio in coronary intermediate lesions.

Background:

Thank you for inviting me to review this important manuscript. The authors have done well in studying a very important subject. They sought to investigate the association between OCT parameters (minimal lumen area and area stenosis) and functionally significant coronary stenosis identified by QFR (QRF 0.80 or less) in patients with intermediate coronary lesions. They found a good correlation between OCT parameters and QRF-determined severe stenosis. I have outlined my comments below.

Comments:

Abstract, introduction

1. Abstract (page 2): The authors should consider adding some baseline characteristics of the participants to the results session.
2. Introduction (page 3): The authors state, “However, the relationship between OCT and coronary stenosis with functional significance under the assessment of QRF has yet to be investigated.” This statement needs a bit more clarity.
3. The authors state, “This study centered on assessing the diagnostic value exhibited by OCT parameters in identifying coronary stenosis with functional significance under the assessment of QRF. This is not clear, and the objective needs to be more specific.

Methods

4. Study design and study population: The authors state, “Date of access to the data for study purposes was 26^th^ August 2020.” – Does this mean they used one day to extract all the data? I think the period for data capturing should be stated.
5. The authors need to be clear on how the patients were selected. Were there any inclusion and exclusion criteria?
6. “This study had been registered in the net of Clinicaltrials.gov” – This needs grammatical correction. They also state the requirement for informed consent was waived. It must be stated which body waived it.
7. There are some grammatical errors in the methodology that need to be corrected.

Results

1. The authors should consider including the participants' sex in the baseline characteristics. What percentage were females?
2. The sample size of 113 patients with 115 lesions is relatively small.
3. The references to the tables and figures did not appear well in the text. The figures should be referred to appropriately in the text, for example, fig (a) on page 9 first line should be fig (4a)
4. Figures and tables: the explanation of the abbreviations should be written alphabetically. In table 3, ‘Ezemab’ should be changed to ezetimibe.
5. The authors state on page 9, paragraph 2, “Upon stenosis being divided according to lesion location in order to assess the diagnostic performance at different vessels, the diagnostic value exhibited by MLA for the identification of significant stenosis for the right coronary artery was heightened.” Rephrasing this will bring some clarity.
6. On page 10, the authors state that OCT was performed for ALL the patients. Meanwhile, 37 patients (40%) were enrolled in the OCT-guided OMT group and 56 patients (60%) in the OCT-guided PCI (OCT-PCI) group. What happened to the rest of the 113 participants?
7. Figure 1 (the flow chart): The authors stated in the methods (page 4) that patients who did not have good angiograms for QRF analysis were excluded from the analysis. However, the figure does not indicate how many patients were excluded based on this reason.

Discussion

1. Some grammatical and typographical errors need to be corrected. Eg ‘ASacquired’ should be ‘AS-acquired’ (page 11, last sentence); OCT-guided PCI instead of OCT-guidance PCI (page 13); FFR-guided PCI instead of FFR-guidance PCI (page 13).
2. The authors need to expand the limitations session, addressing, for example, the small sample size, confounders, and generalizability.

Acknowledgement – Please change ‘my’ to ‘our’.
